# Miniature Switchable Millimeter-Wave BiCMOS Low-Noise Amplifier at 120/140 GHz Using an HBT Switch

**DOI:** 10.3390/mi10100632

**Published:** 2019-09-21

**Authors:** Julio Heredia, Miquel Ribó, Lluís Pradell, Selin Tolunay Wipf, Alexander Göritz, Matthias Wietstruck, Christian Wipf, Mehmet Kaynak

**Affiliations:** 1 Universitat Politècnica de Catalunya, Campus Nord UPC mòdul D3, 08034 Barcelona, Catalonia, Spain; 2 La Salle—Universitat Ramon Llull, 08022 Barcelona, Catalonia, Spain; 3 IHP—Leibniz-Institut für innovative Mikroelektronik, 15236 Frankfurt (Oder), Germany

**Keywords:** low-noise amplifier (LNA), frequency-reconfigurable LNA, multimodal circuit, SiGe BiCMOS, hetero junction bipolar transistor (HBT), RF switch

## Abstract

A 120–140 GHz frequency-switchable, very compact low-noise amplifier (LNA) fabricated in a 0.13 µm SiGe:C BiCMOS technology is proposed. A single radio-frequency (RF) switch composed of three parallel hetero junction bipolar transistors (HBTs) in a common-collector configuration and a multimodal three-line microstrip structure in the input matching network are used to obtain a LNA chip of miniaturized size. A systematic design procedure is applied to obtain a perfectly balanced gain and noise figure in both frequency states (120 GHz and 140 GHz). The measured gain and noise figure are 14.2/14.2 dB and 8.2/8.2 dB at 120/140 GHz respectively, in very good agreement with circuit/electromagnetic co-simulations. The LNA chip and core areas are 0.197 mm^2^ and 0.091 mm^2^, respectively, which supposes an area reduction of 23.4% and 15.2% compared to other LNAs reported in this frequency band. The experimental results validate the design procedure and its analysis.

## 1. Introduction

The D-band (110–170 GHz) millimeter-wave (mm-wave) frequency region is attracting a growing interest for the development of future short-distance, high-speed line-of-sight fixed-communication systems [1,2]. The International Telecommunication Union (ITU) radio regulations [3] define frequency allocations for those services in the frequency ranges 122.25–123 GHz and 141–148.5 GHz. Applications such as backhaul, front-haul and fixed wireless access in point-to-point or point-to-multipoint systems, 5G mobile backhaul tail link and inter-server connection systems have been envisaged, and several demonstration prototypes of D-band links in the frequency range 125–160 GHz have been reported [4]. A multiband architecture is often required for an efficient use of the spectrum [5,6] and, in order to enable inexpensive and versatile communication systems, frequency reconfigurable devices, in particular low-noise amplifiers (LNAs) are highly desirable. This way the size, cost, and power consumption are minimized.

The SiGe BiCMOS technology has demonstrated maturity and high performance for D-band wireless systems and sensors [2,7,8]. Recent advances in 0.13 µm SiGe BiCMOS hetero junction bipolar transistor (HBT) devices [9] have enabled the development of fixed-frequency LNAs using two to four HBT stages, typically in a cascode configuration [1,10,11,12,13,14,15,16,17], featuring a high gain *G* (*G* = 24 dB at 158 GHz [1]) and low noise figure *F* (*F* = 5.1 dB at 144.5 GHz [14]). Other silicon technologies, such as 65- and 28-nm SOI CMOS, are also being used to fabricate D-band LNAs [18,19,20], featuring comparable gain but a higher noise figure (*G* = 15.7 dB and *F* = 8.5 dB at 160 GHz [20]). Regarding frequency-reconfigurable mm-wave LNAs, only a few have been reported to date, at 60/77 GHz [15], 24/79 GHz [16], and at D-band (125/140 GHz) [17], all using the SiGe BiCMOS process. The frequency reconfiguration is provided with radio-frequency microelectromechanical system (RF-MEMS) switches (elements included in the design-kit library [8]). Though the design in [17] was not optimized for a maximal *G* or minimal *F*, but for a balanced *G* and *F* in the two frequency states, it features a gain *G* = 18.2/16.1 dB and noise figure *F* = 7/7.7 dB at 125/140 GHz, which compare well with those of fixed-frequency LNAs [1,11,12] and are competitive for the intended applications.

Regarding miniaturization, the frequency-reconfigurable D-band LNA presented in [17] exhibits both, the smallest chip area, *A*_CHIP_, and core area (without RF- and DC-pads), *A*_CORE_, compared to the other D-band LNAs [1,10,11,12,13,14]. In [17], the RF-MEMS switch area is 0.031 mm^2^, which supposes a 29% of *A*_CORE_ (*A*_CORE_ = 0.107 mm^2^). Since the RF-MEMS switch dimensions barely scale with frequency (0.040 mm^2^ for devices in the 60–110 GHz [15] vs. 0.031 mm^2^ for devices in the 110–170 GHz frequency band [17]), it is pertinent to explore other options to design more compact RF switches. One potential application is future frequency-reconfigurable BiCMOS LNAs at higher frequencies, such as the G-band (140–220 GHz) and WR-4 waveguide band (170–260 GHz), at which the RF-MEMS switch area may become comparable to the core area. As an example, the (estimated) *A*_CORE_ of the 245 GHz fixed-frequency LNA reported in [21] is only 0.036 mm^2^.

In this paper a frequency-reconfigurable BiCMOS LNA at 120/140 GHz is presented. The LNA design is based on the one reported by the authors in [17] but now an HBT-based switch is used instead of a RF-MEMS switch. As a result, the LNA size is further reduced with only a slight decrease in gain and increase in noise figure, but with enhanced gain balance and noise-figure balance in both frequency states. The paper is organized as follows: In Section 2 the LNA design and HBT-based switch design are presented, and the HBT-switch performances are compared to that of the RF-MEMS switch. In Section 3 the experimental results are presented, discussed, and compared to previous works. Finally, Section 4 concludes the paper and its contributions are highlighted.

## 2. Design of the Frequency-Reconfigurable LNA 

**Figure 1:**
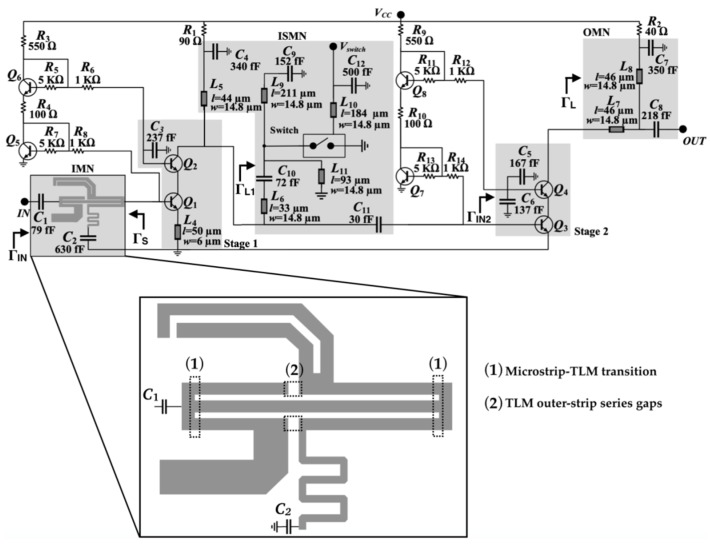
Schematic of the switchable BiCMOS low-noise amplifier (LNA) at 120/140 GHz and a detail of the multimodal three-line-microstrip (TLM) structure implemented in the input matching networks (IMN).

The LNA schematic is shown in Figure 1. It consists of two cascode stages. The first stage is composed of HBTs *Q*_1_ and *Q*_2_ and the second stage of HBTs *Q*_3_ and *Q*_4_. They are biased using current mirrors (*Q*_5_/*Q*_6_ and *Q*_7_/*Q*_8_, respectively) and bias resistors (*R*_1_ and *R*_2_). Similarly to [17], the input and output matching networks (IMN and OMN, respectively) are fixed, and the frequency-reconfiguration capability is only introduced in the LNA inter-stage matching network (ISMN), to reduce the design complexity, size, and weight.

**Figure 2:**
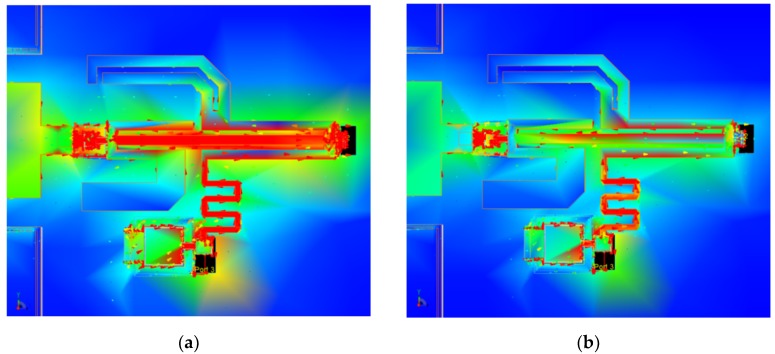
IMN electromagnetic (EM) simulation results. Simulated current distribution on the IMN TLM multimodal structure. (**a**) 120 GHz. (**b**) 140 GHz.

The IMN is based on a compact three-line-microstrip (TLM) section connected to input/output short microstrip lines, with two central series gaps in its outer strips [17]. The TLM simultaneously propagates three fundamental modes: even-even (*ee*), odd-odd (*oo*), and odd-even (*oe*) [22]. The microstrip modes basically generate *ee* modes at the microstrip-to-TLM transitions, which then excite (and afterwards interact with) the *oo* and *oe* modes at the gaps, which in turn resonate in the TLM section. The interaction between all these modes results in an IMN featuring a large electrical size with reduced physical area. To illustrate the modal conversion as well as the resonance condition in the TLM multimodal structure, Figure 2 shows the simulated current density distribution on the metallization for the two frequency states (120 GHz and 140 GHz). The simulation was performed using the electromagnetic (EM) simulator Momentum 2.5D (Keysight Technologies, Santa Rosa, CA, USA). The (symmetrical) *ee* mode features equally-oriented currents on the three TLM strips, the (symmetrical) *oo* mode features equally-oriented currents in the outer strips and reverse-oriented current in the center strip, and the (anti-symmetrical) *oe* mode features reverse-oriented currents in the outer strips and no current in the center strip [19]. The anti-symmetrical *oe* mode can only be generated in the asymmetrically loaded gaps in the outer TLM strips, whereas the symmetrical *ee* and *oo* modes can, in addition, be generated at the symmetrical transitions to microstrip lines at both ports of the IMN. The high levels of currents in the center strip for the 120-GHz state indicate the predominance of the *ee* and *oo* modes for the performance of the IMN in this state, whereas the markedly asymmetrical current distribution for the 140-GHz state indicates the predominance of *oo* and *oe* modes in the performance of the IMN in this state. The characteristic impedances and propagation constants of each TLM-fundamental mode were first determined from electromagnetic simulation of a basic TLM section without any discontinuity. Then the dimensions (lengths, strip widths, and gaps) of the TLM structure in the IMN were optimized by using modal equivalent circuits which transform the actual voltages and currents on generic *n*-conductor lines (in this case, *n* = 3) into their modal counterparts [23]. This way the IMN physical dimensions were minimized to obtain a structure of reduced size designed to combine a low LNA noise figure (*F*) and a small input-reflection coefficient magnitude |Γ*_IN_*|, both balanced at the two frequency states (120/140 GHz).

**Figure 3:**
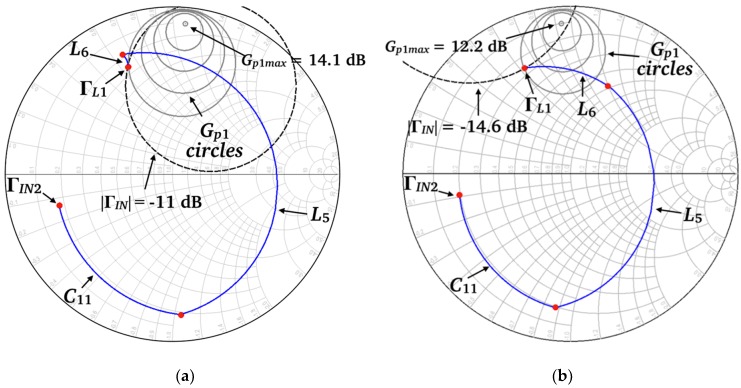
Inter-stage matching network (ISMN) design and plots of constant *G_p_*_1_ circles and constant *F* and |Γ*_IN_*| circles on the Γ*_L_*_1_ plane. (**a**) 120 GHz frequency state; (**b**) 140 GHz frequency state.

The OMN is composed of a simple line-short-circuited stub structure (microstrip lines *L*_7_ and *L*_8_), designed to attain a balanced second-stage power gain *G_p_*_2_ at both frequency states. The ISMN consists of a two-segment short-circuited stub *L*_6_/*L*_9_, a second short-circuited stub *L*_5_, an RF switch terminated with a short-circuited stub *L*_11_, biased through a λ⁄4 stub (*L*_10_), and a series capacitor *C*_11_. *C*_9_, *C*_10_, and *C*_12_ are decoupling capacitors. The purpose of *L*_5_ is to allow a shorter *L*_6_ segment, thus reducing the chip transversal dimension. The RF switch selects the two-segment stub length, either *L*_6_ when the switch is in its ON state (for the high-frequency, 140 GHz LNA state), or *L*_6_ + *L*_9_ when the switch is in its OFF state (for the low-frequency, 120 GHz LNA state). As shown in Figure 3, the ISMN line lengths and the series capacitor value *C*_11_ were designed to present Γ*_L_*_1_ values that guarantee a first-stage power gain *G_p_*_1_ balanced at both frequency states (*G_p_*_1_ = 9.6/8.9 dB at 120/140 GHz). Therefore, the combined effect of the OMN and the ISMN is to balance the LNA power gain *G_p_* (*G_p_* = *G_p_*_1_·*G_p_*_2_) at both frequency states. Those Γ*_L_*_1_ values simultaneously fulfill the IMN requirements for a low and balanced *F* and |Γ*_IN_*|. This can graphically be assessed by plotting on the Γ*_L_*_1_ plane the geometrical locus of constant *F* and |Γ*_IN_*| (for a given source reflection coefficient), which is a circle. These circles, for |Γ*_IN_*| = −11/−14.6 dB and *F* = 6.1/6.5 dB at 120/140 GHz, are also plotted in Figure 3. It can be observed that the circles intersect the intended Γ*_L_*_1_ values, and in consequence the requirements for *G_p_*, *F*, and |Γ*_IN_*| at both frequency states are satisfied simultaneously. The simulated results were obtained from circuit/electromagnetic (EM) co-simulation, using manufacturer circuit models for HBTs (including the transistors used in the RF switch) and resistors, and electromagnetic models (obtained from electromagnetic simulation) for passives (lines, metallization on different layers, pads, via-holes, capacitors and ground plane). The simulation platform was Keysight ADS/2.5D Momentum. From the electromagnetic analysis an electromagnetic model (co-simulation element) was obtained, which was used in a circuit simulation as a block to which the manufacturer’s design kit models of HBTs and resistors were connected.

**Figure 4:**
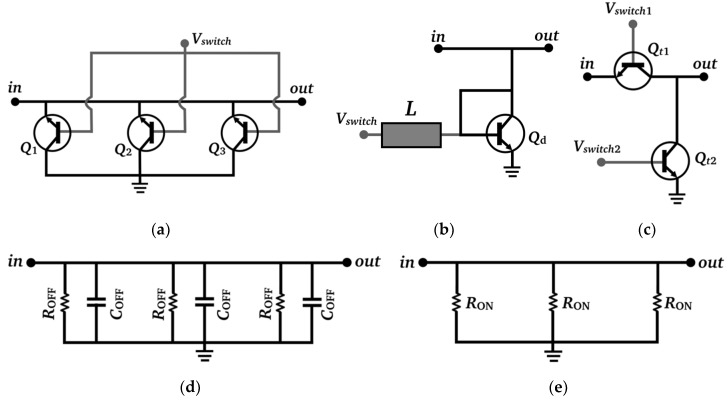
Hetero junction bipolar transistor (HBT) radio frequency (RF) switch. (**a**) Selected witch configuration (shunt-connected HBTs in reverse saturation mode). The switch activation voltage is *V_SWITCH_* = 1 V. Other switch configurations: (**b**) Diode configuration; (**c**) L-shape configuration. Equivalent circuit of the selected switch (**a**): (**d**) OFF state (*C_OFF_* = 7.2 fF, *R_OFF_* = 1600 Ω ); (**e**) ON state (*R_ON_* = 10 Ω).

For the RF switch, three different configurations were considered (shown in Figure 4a–c). The shunt-HBT configuration (Figure 4a) uses three shunt-connected HBTs in reverse-saturation mode. The diode configuration (Figure 4b) uses one or two shunt-connected HBTs as diodes. The L-shape configuration (Figure 4c) uses two HBTs, series- and shunt-connected respectively. Preliminary simulations showed that the L-shape and shunt-HBT configurations had similar ON-state isolation but the L-shape OFF-state insertion loss was much higher. The diode configuration showed poorer ON-state isolation and OFF-state insertion loss than the shunt-HBT configuration. Therefore, the L-shape and diode configurations were dismissed as an option for the RF switch, and the selected configuration was the shunt-HBT in reverse-saturation mode (Figure 4a). The three shunt HBTs (*Q*_1_, *Q*_2_, and *Q*_3_ in Figure 4a) are in a common-collector configuration. With reference to Figure 1, the emitters are connected to *L*_11_, the collectors are connected to ground and the switch bias voltage *V_SWITCH_* is supplied through the λ⁄4 stub *L*_10_. When *V_SWITCH_* = 0 V (Figure 4b), the transistors are in the cut-off region mode and they behave as a high impedance *R_OFF_* in parallel with a parasitic capacitance *C_OFF_* (the HBT-based RF switch is in OFF state). *L*_11_ adds an inductance which resonates with the *C_OFF_* capacitances, thus reducing their effect on the switch. When *V_SWITCH_* = 1 V (Figure 4c), the transistors are in the saturation region mode and they behave as a low impedance *R_ON_* (the HBT-based RF switch is in ON state). Connecting the switch HBTs in reverse saturation mode (the HBTs are flipped) isolates the emitter from the silicon substrate, thus reducing the parasitic capacitances; also, the impedance in OFF state is larger since the potential barrier in the conduction band is larger at the emitter than at the collector [24]. The RF-switch DC-power consumption in ON state is 15 mW. The number of transistors used for the selected shunt-HBT configuration was a trade-off between performance and power consumption. If two HBTs were used, the DC-power consumption would decrease to 12 mW but the ON-state isolation would worsen (15.5 dB). Using four HBTs would increase the ON-state isolation to 20.8 dB but the OFF-state insertion loss and power consumption would increase to 0.46 dB and 24 mW, respectively.

**Figure 5:**
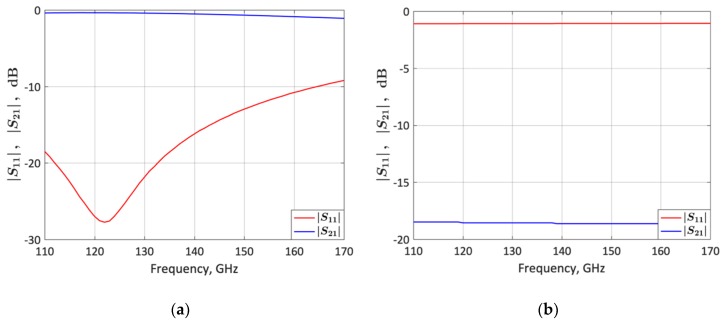
Simulated *S*-parameters of the HBT-RF switch of Figure 4a (designed on SG13G2 0.13 µm SiGe:C BiCMOS technology) taking into consideration the stubs *L*_10_ and *L*_11_ connected to the HBT base and emitter, respectively, as shown in Figure 1. (**a**) Switch in OFF state. (**b**) Switch in ON state.

Figure 5 plots simulations of the proposed HBT-switch *S*-parameters for the configuration shown in Figure 4a, taking into consideration the stubs *L*_10_ and *L*_11_ connected to the HBT base and emitter, respectively, as shown in Figure 1. From Figure 5, the calculated switch-insertion loss (*IL*), defined as *IL* = −10·log(|*S*_21_(OFF)|^2^), is 0.36 dB at 120 GHz, and the calculated switch isolation (*I*), defined as *I* = −10·log(|*S*_21_(ON)|^2^), is 18.6 dB at 140 GHz. The stub length *L*_11_ was tuned to resonate with *C_OFF_* to minimize *IL* at 120 GHz. The RF-MEMS switch used in [17] features *IL* = 0.25 dB at 120 GHz and *I* = 32 dB at 140 GHz [8]. Therefore, the proposed HBT switch presents a lower isolation and a higher insertion loss than its RF-MEMS switch counterpart. The performance trade-off between MEMS and HBT switches for the LNA design can be observed by comparing Figure 3 with Figure 2 of [17]. *G_p_*_1_ decreases and the noise figure increases in both frequency states, but the decrease in *G_p_*_1_ is smaller in the high-frequency state (1.6/1 dB respectively). Thus, as discussed in Section 3, it is possible to get a perfectly balanced gain and noise figure between both frequency states, and the chip size is substantially reduced.

**Figure 6:**
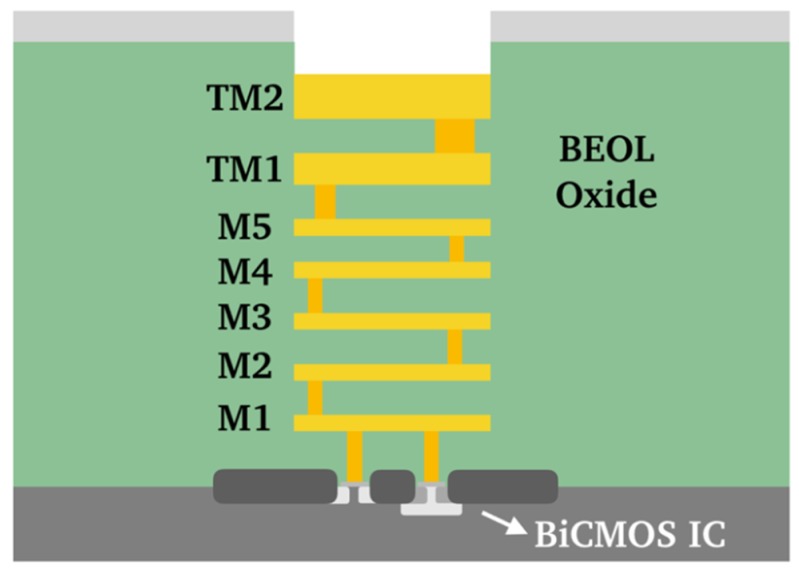
Cross section of the back-end-of-line (BEOL) in the SG13G2 0.13 µm SiGe:C BiCMOS technology from IHP—Leibniz-Institut für innovative Mikroelektronik [25].

The LNA was fabricated in SG13G2 0.13 µm SiGe:C BiCMOS technology using HBTs with *f_T_* / *f_max_* of 300/500 GHz and 0.9 µm emitter length [25] from IHP—Leibniz-Institut für innovative Mikroelektronik. The back-end-of-line (BEOL) consists of five metal layers (M1–M5) and two top-metal layers, TM1, and TM2 (Figure 6). All lines on the ISMN (*L*_5_, *L*_6_, *L*_9_, *L*_10_, and *L*_11_) and OMN (*L*_7_ and *L*_8_,), as well as line *L*_4_ are microstrip lines. The top-most metal layer TM2 was used for *L*_5_, *L*_6_, *L*_7_, *L*_8_ *L*_10_, *L*_11_, and the multimodal TLM structure of the IMN. *L*_9_ was fabricated using the TM1 layer, and *L*_4_ with a stack of three layers (M2, M3, and M4). The calculated *Q* factor from EM simulation is 37 for both frequencies (120 GHz and 140 GHz). The number of emitter fingers for transistors *Q*_1_/*Q*_2_/*Q*_3_/*Q*_4_ in stages 1 and 2 of the cascode configuration (Figure 1) is 5/10/10/10. This combination was selected according to the gain and noise figure required for each stage, while keeping a low DC-power consumption. The number of emitter fingers of each transistor in the HBT-RF switch is 7, which assures the required current flows for the transistors in ON state (*I_B_* = 5 mA for each transistor, *I_C_* = 1.9/1.7/1.7 mA for *Q*_1_/*Q*_2_/*Q*_3_ in Figure 4a). The LNA total DC-power consumption *P_DC_*, including the two stages and the RF switch is *P_DC_* = 37.5 mW for the 120 GHz frequency state and *P_DC_* = 52.5 mW for the 140 GHz frequency state. 

**Figure 7:**
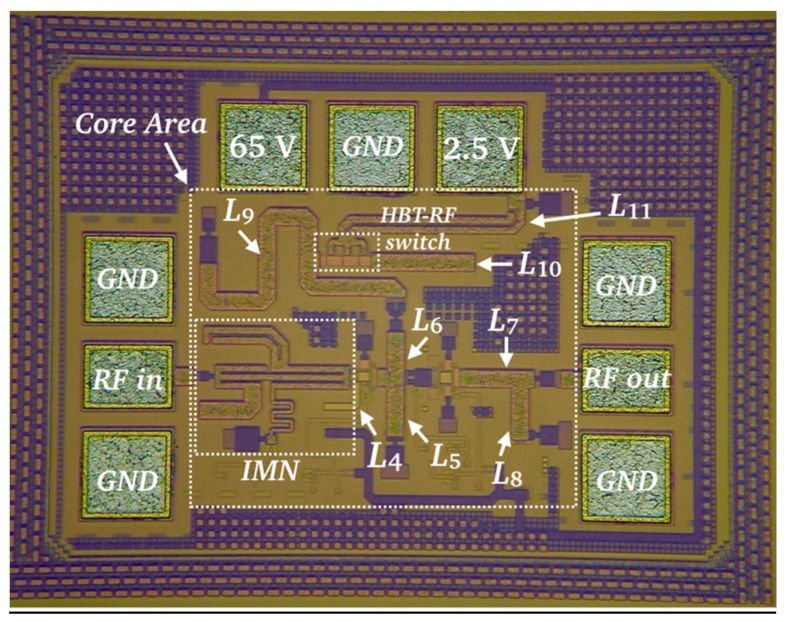
Micrograph of the frequency-switchable LNA including and HBT-RF switch fabricated in a SG13G2 0.13 µm SiGe:C BiCMOS technology from IHP [25]. Dimensions are: *A_CHIP_* = 515 µm × 382 µm and *A_CORE_* = 331 µm × 274 µm. The HBT-RF switch area is: 45.4 µm × 23.6 µm.

Figure 7 shows a micrograph of the fabricated LNA. The LNA features an HBT-switch area of 45.4 µm × 23.6 µm, which is much smaller than the RF-MEMS switch area (260 µm × 118 µm) of the previous LNA presented in [17]. The chip and core areas are *A_CHIP_* = 515 µm × 382 µm and *A_CORE_* = 331 µm × 274 µm, which supposes an area reduction of 23.4% and 15.2%, respectively, compared to [17].

## 3. Results and Discussion

### 3.1. S-Parameter Simulation and Measurement

**Figure 8:**
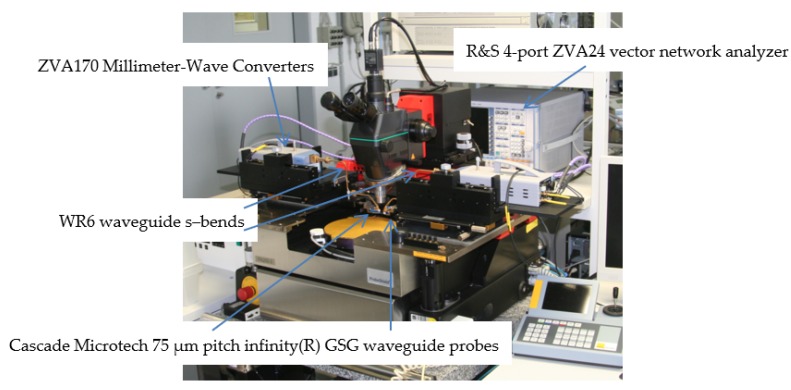
110–170 GHz S-parameter measurement setup using a semi-automated wafer probe station.

**Figure 9:**
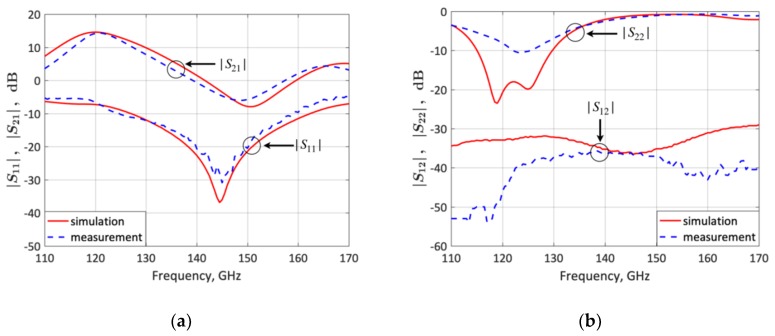
Measured and simulated LNA *S*-parameters for the lower-frequency state (120 GHz). (**a**) *S*_21_ and *S*_11_; (**b**) *S*_12_ and *S*_22_.

**Figure 10:**
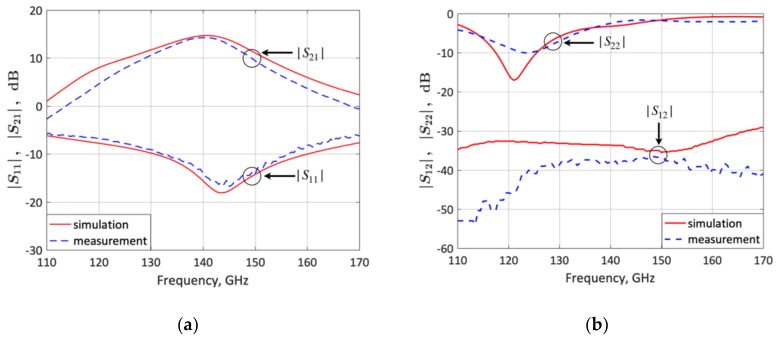
Measured and simulated LNA S-parameters for the upper-frequency state (140 GHz). (**a**) *S*_21_ and *S*_11_; (**b**) *S*_12_ and *S*_22_.

The *S*-parameters of the fabricated frequency-switchable LNA were experimentally characterized from 110 to 170 GHz on a semi-automated wafer probe station with a setup from Rohde & Schwarz, consisting of a 4 port ZVA24 as vector network analyzer and two ZVA170 millimeter-wave converters (Figure 8). The Cascade Microtech 75 µm-pitch infinity(R) GSG waveguide probes were connected via WR6 waveguide s–bends to the millimeter–wave converters. For the calibration, the impedance standard substrate (ISS 138–356) was placed together with an RF absorber on an auxiliary ceramic chuck and a full two-port LRRM calibration was performed. The applied bias voltage *V_CC_* (Figure 1) was *V_CC_* = 2.5 V and the input RF power was −20 dBm. Figure 9 and Figure 10 compare the measured and simulated LNA *S*-parameters for the lower-frequency state (120 GHz) and upper-frequency state (140 GHz), respectively. The LNA features a measured |*S*_21_|, |*S*_11_|, |*S*_12_|, and |*S*_22_| of 14.2, −6.6, −46, and −8.1 dB respectively for the lower-frequency state, and 14.2, −14, −37.9, and −2.5 dB respectively for the upper-frequency state. These results are in good agreement with simulations.

### 3.2. Noise-Figure (F) Simulation and Measurement

The noise figure *F* was measured for both frequency states. The measurement was also carried out on wafer using the *Y*-factor method. The measurement setup is described in [20]. Hot and cold noise temperatures are produced by a noise diode Elva-1 ISSN-06. The noise power is down-converted to a 50 MHz IF using a subharmonic mixer with amplifier-multiplier chain as LO (MixAMC-192, Virginia Diodes Inc., Charlottesville, VA, USA), and measured using a noise figure analyzer Agilent N8973A (now Keysight Santa Rosa, CA, USA).

**Figure 11:**
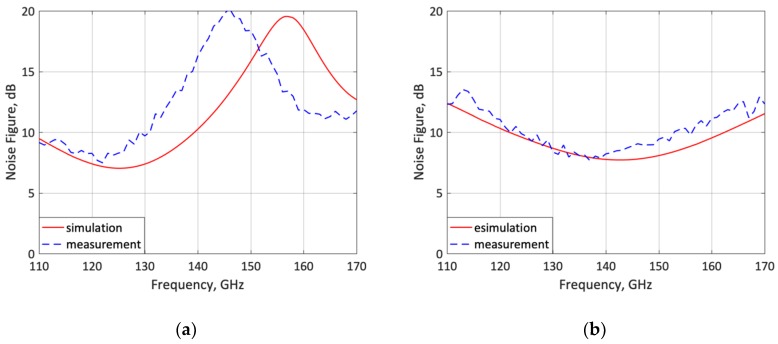
Measured and simulated LNA noise figure. (**a**) Lower-frequency state (120 GHz); (**b**) upper-frequency state (140 GHz).

The simulated *F* is compared to the measured results for the lower- and upper-frequency states in Figure 11. The measured *F* is 8.2 dB for both lower- and upper-frequency states, in close agreement with simulation.

The noise-figure peak shift in low-frequency state is attributed to the EM simulation which underestimates the small inductance associated to the metallization and via holes connecting each transistor emitter of the HBT switch.

### 3.3. Stability (µ-Factor) Simulation and Measurement

**Figure 12:**
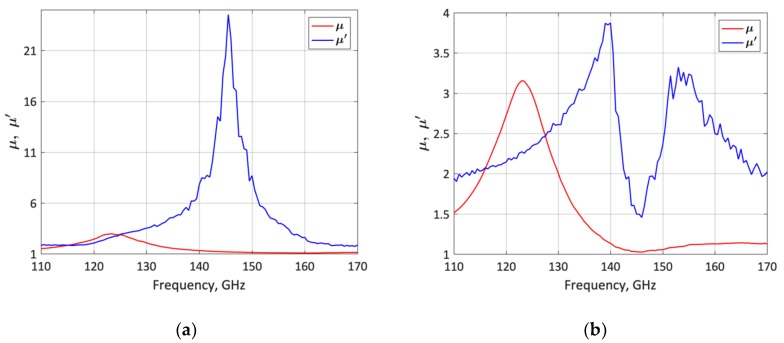
Stability factors (*µ* and *µ*’) obtained from the measured *S*-parameters. (**a**) Lower-frequency state (120 GHz); (**b**) upper-frequency state (140 GHz).

The stability of the LNA was assessed using the *µ* and *µ*’ factors [26]. According to the simulations performed from DC to 170 GHz, the LNA is unconditionally stable in all frequencies (*µ* > 1 and *µ*’ > 1) for both lower- and upper-frequency states. The *µ* and *µ*’ stability factors were also obtained from the measured results demonstrating that the LNA is unconditionally stable in the 110 to 170 GHz frequency band for both frequency states. As shown in Figure 12, the minimal calculated *µ* and *µ*’ values obtained from the measurements are *µ* = 1.07 and *µ*’ = 1.82 for the lower- frequency state, and *µ* = 1.02 and *µ*’ = 1.45 for the upper-frequency state.

### 3.4. Simulated 1-dB Gain Compression Point (P_1dB_)

**Figure 13:**
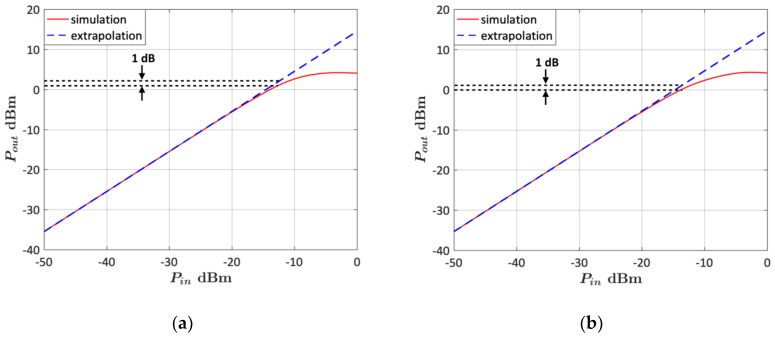
Non-linear simulation of the LNA output power vs. input power. (**a**) Lower-frequency state (120 GHz); (**b**) upper-frequency state (140 GHz).

The 1-dB gain compression point (*P*_1*dB*_) was obtained from a non-linear simulation of the LNA. The LNA input power was swept from −50 dBm to 0 dBm and the output power was simulated using the HBT non-linear model provided by the manufacturer. In Figure 13, the simulated output power is plotted vs. the input power and compared to the theoretical small-signal output power. It can be observed that the input-referred *P*_1*dB*_ is −12.4 dBm for the lower-frequency state and−13.6 dBm for the upper-frequency state.

### 3.5. Discussion of Results

The LNA S-parameters and *F* measurements presented in Figure 9, Figure 10 and Figure 11 are in good agreement with the simulations and well balanced at both frequency states. Indeed, it is observed that the gain (|*S*_21_|) and noise figure *F* feature the same measured values (14.2/14.2 dB and 8.2/8.2 dB) at 120/140 GHz, respectively. Thus, the frequency-reconfigurable LNA concept and design methodology is validated. Table 1 shows a comparison of the LNA measured parameters and those of other reconfigurable and not-reconfigurable cascaded SiGe BiCMOS mm-wave LNAs reported in the literature. In order to evaluate and compare the LNA performance, a figure-of-merit (FoM) is defined as

(1) $$FoM=\;\frac{1000\;\times \;G\;\cdot P_{1dB}\;}{(F-1)\cdot P_{DC}\cdot A}$$

where *G* is the LNA gain (*G* = |*S*_21_|^2^) and the area *A* refers either to *A_CHIP_* or *A_CORE_*. 

The proposed LNA exhibits the smallest area *A* (both *A_CHIP_* and *A_CORE_*) and the highest FoM (save [11] and [17] at 143 GHz with a similar FoM). Compared to the frequency-reconfigurable LNAs based on RF-MEMS switches [15,16,17], it is more compact at the expense of a lower gain and higher *P_DC_* in the upper-frequency state, and it exhibits the same (or very similar) *F* at comparable frequencies. Though the LNA was not designed for a maximal *G* or minimal *F*, but for a balanced *G* and *F* in the two frequency states, its *F* is comparable or better than [1,12] at similar frequencies, which are not reconfigurable and were optimized for low-noise performance.

## 4. Conclusions

A 120–140 GHz frequency-reconfigurable 0.13 µm SiGe:C BiCMOS, very-compact D-band LNA has been presented. A single HBT switch is used in the inter-stage matching network to minimize size and design complexity. The HBT switch is composed of three transistors in parallel, featuring 0.36 dB switch-insertion loss in OFF state and 18.6 dB switch-isolation in ON state. The LNA size is minimized by using, in addition to a single HBT switch, a multimodal three-line-microstrip input-matching network. A systematic general procedure has been applied to design the input-, inter-stage- and output-matching networks to obtain a perfectly balanced gain and noise figure at both frequency states. The measured gain and noise figure are 14.2/14.2 dB and 8.2/8.2 dB at 120/140 GHz, respectively, in very good agreement with circuit/electromagnetic co-simulations. The chip and core areas (0.197/0.091 mm^2^) are the smallest reported in the literature in this frequency band. The experimental results validate the design procedure and its analysis, and prove that reconfigurable devices based on HBTs can be a viable alternative to those based on MEMS switches whenever the performance specifications are not exceedingly demanding and compactness can be an issue (e.g., in space applications), besides saving cost and fabrication steps.

## Figures and Tables

**Table 1:** Comparison with other cascaded SiGe BICMOS mm-wave LNAs.

	Technology (µm)	Frequency (GHz)	*G* (dB)	*P*_1*dB*_ (dBm)	*F* (dB)	*P_DC_* (mW)	*A_CHIP_* / *A_CORE_* (mm^2^)	FoM
[1] †	0.13	158	24.1	−25.9	8.2	28	0.342/0.18^††^	12.31/23.38
[10]	0.13	140	23.3	−33**	5.5	12	0.393/0.231^††^	8.92/15.17
[11]	0.13	130	24.3	−17.3	6.8	84	0.301/0.192^††^	52.35/82.07
[12]	0.13	145	21		8.5	14.5	0.36/0.270^††^	
[13]	0.09	140	30		6.2	45	0.525/0.115	
[14]	0.13	144.5	32.6	−37.6	5.1++	28	1/0.6	5.05/8.42
[15]	0.25	60	20*	−18**	7*	40	0.788/0.317^††^	12.53/31.2
[15]	0.25	77	22*	−18**	8*	40	0.788/0.317^††^	15.01/37.31
[16]	0.25	24	25	−27**	4.3**	40	0.770/0.476^††^	12.11/19.6
[16]	0.25	74	18	−18**	8.5**	40	0.770/0.476^††^	5.34/8.63
[17]	0.13	125	18.2	−17.3**	7	36.8	0.257/0.107	32.42/78.17
[17]	0.13	143	16.1	−15.9**	7.7	36.8	0.257/0.107	22.65/54.6
**This**	**0.13**	**120**	**14.2**	−12.4**	**8.2**	**37.5**	**0.197/0.091**	**37.34/80.99**
**This**	**0.13**	**140**	**14.2**	−13.6**	**8.2**	**52.5**	**0.197/0.091**	**20.12/43.63**

† Cascode; * Averaged; ** Simulated; ++ Estimated.
